# Supplementary material for: Mesoporous CuO Prepared in a Natural Deep Eutectic Solvent Medium for Effective Photodegradation of Rhodamine B
Source: Molecules. 2023 Jul 20;28(14):5554. doi: 10.3390/molecules28145554 (PMC10384838; doi:10.3390/molecules28145554)
Supplement: Supplementary file 1 [file molecules-28-05554-s001.zip › molecules-2465917-supplementary.pdf]

Mesoporous CuO Prepared in a Natural Deep Eutectic Solvent Medium for Effective Photodegradation of Rhodamine B

Sheli Zhang[1], Yuerong Yan[1], Wei Hu[2] and Yunchang Fan[2,]*

[1]College of Science and Technology, Jiaozuo Teachers College, Jiaozuo 454000, China; 1295006035@jzsz.edu.cn (S.Z.); 1295002321@jzsz.edu.cn (Y.Y.)

[2]College of Chemistry and Chemical Engineering, Henan Polytechnic University, Jiaozuo 454003, China; fanyunchang@hpu.edu.cn (Y.F.); huwei@home.hpu.edu.cn (W.H.)

*Correspondence: fanyunchang@hpu.edu.cn (Y.F.)

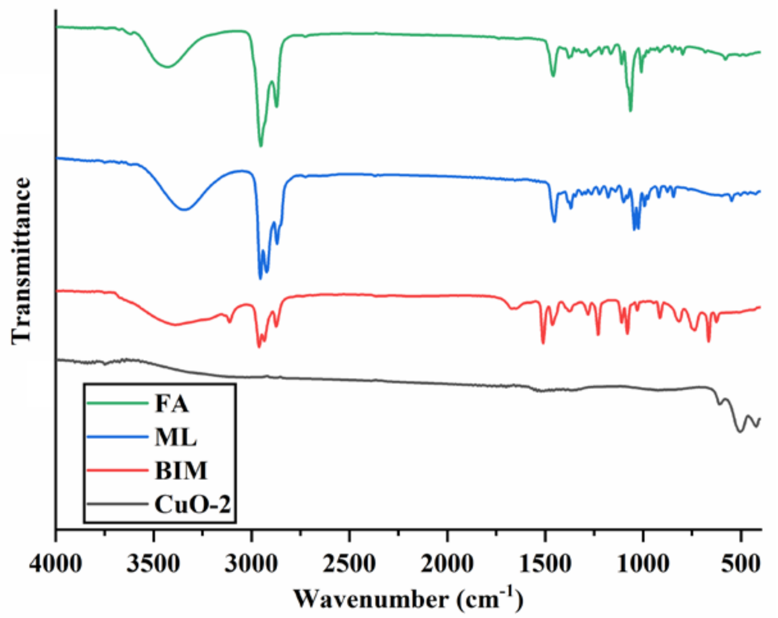

Figure S1. The FT-IR spectra of FA, ML, BIM and CuO-2 NPs.

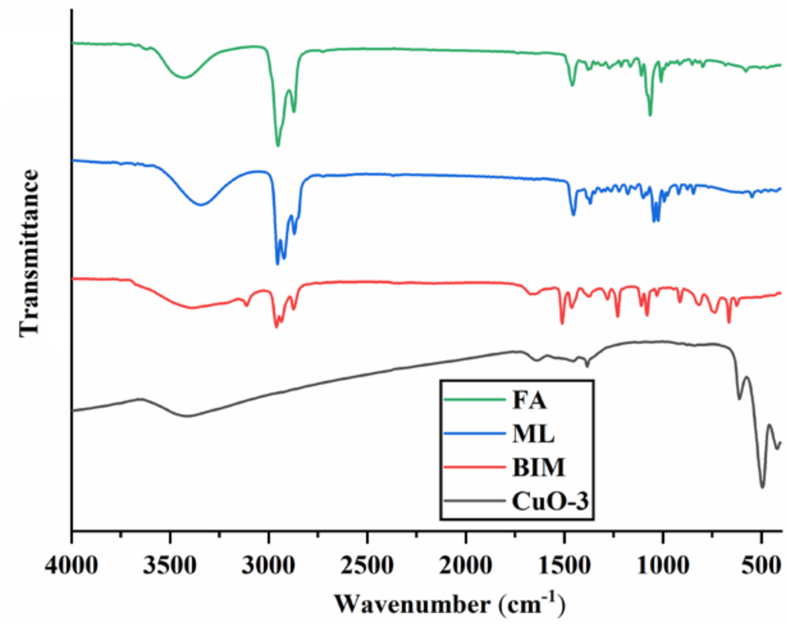

Figure S2. The FT-IR spectra of FA, ML, BIM and CuO-3 NPs.

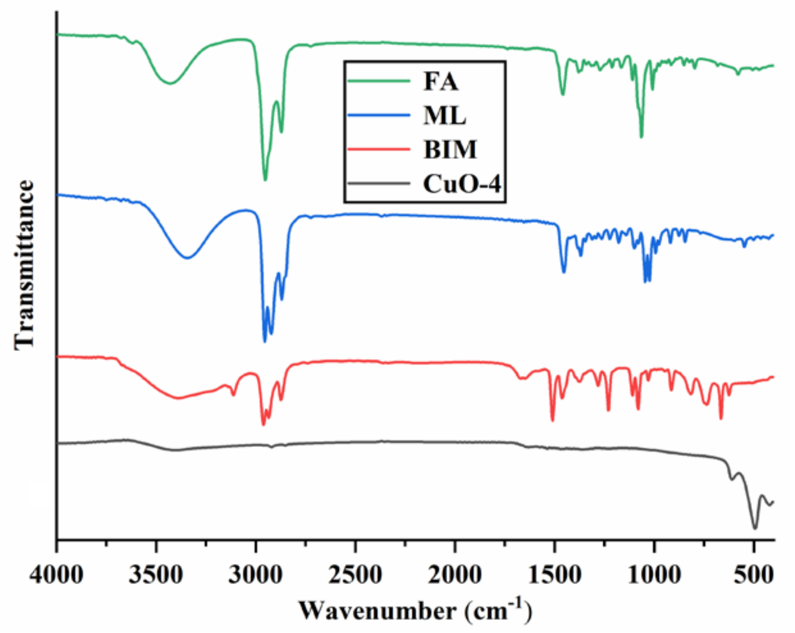

Figure S3. The FT-IR spectra of FA, ML, BIM and CuO-4 NPs.

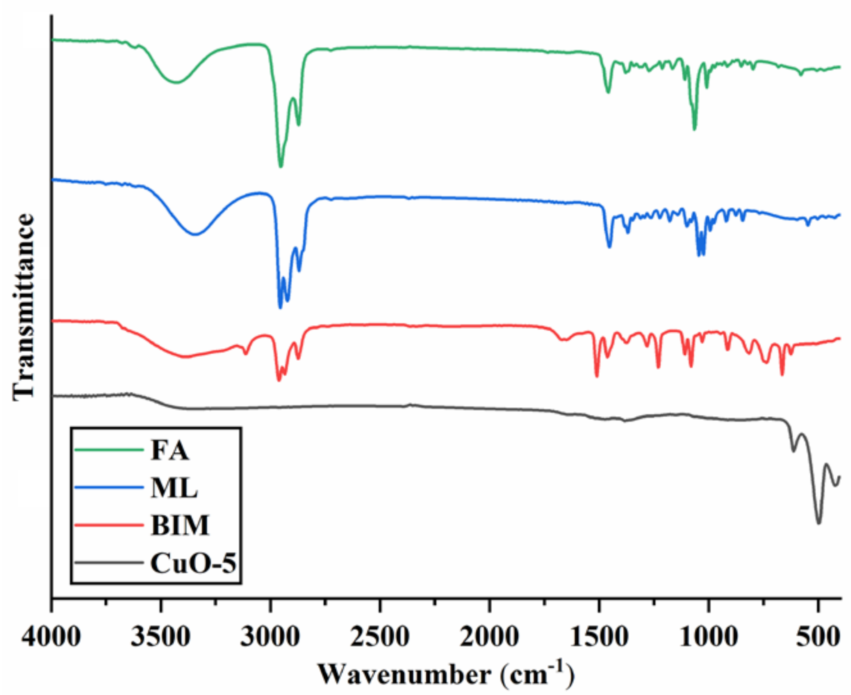

Figure S4. The FT-IR spectra of FA, ML, BIM and CuO-5 NPs.

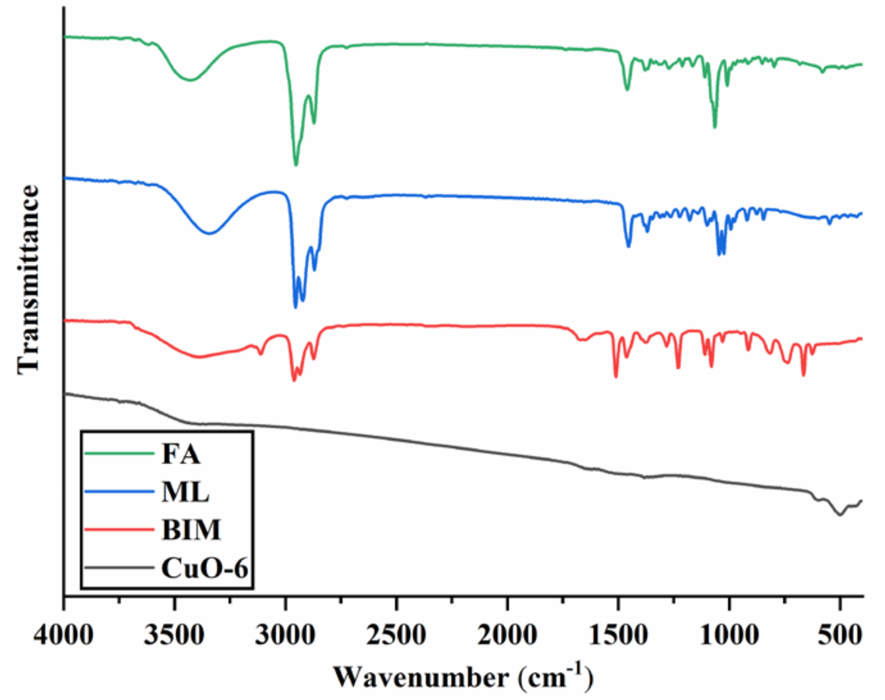

Figure S5. The FT-IR spectra of FA, ML, BIM and CuO-6 NPs.

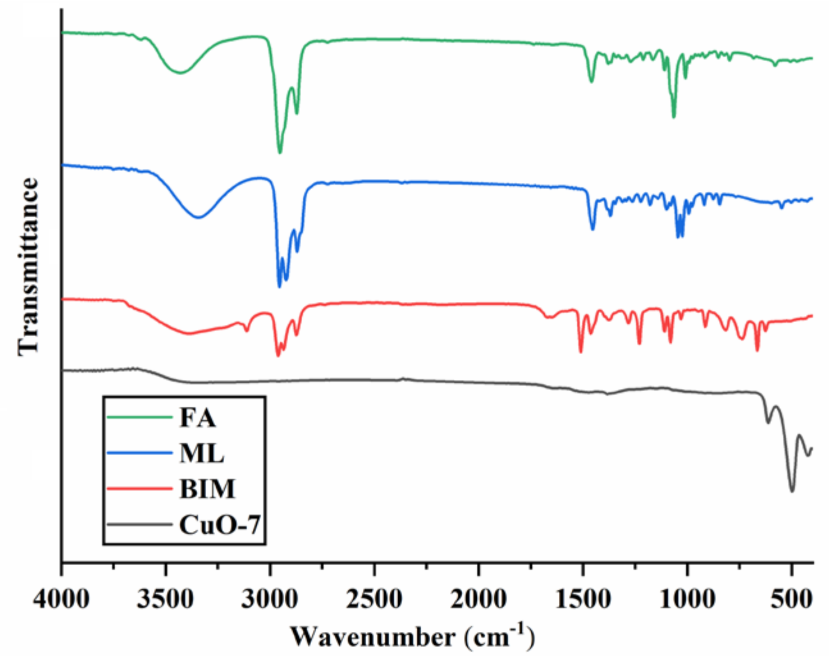

Figure S6. The FT-IR spectra of FA, ML, BIM and CuO-7 NPs.

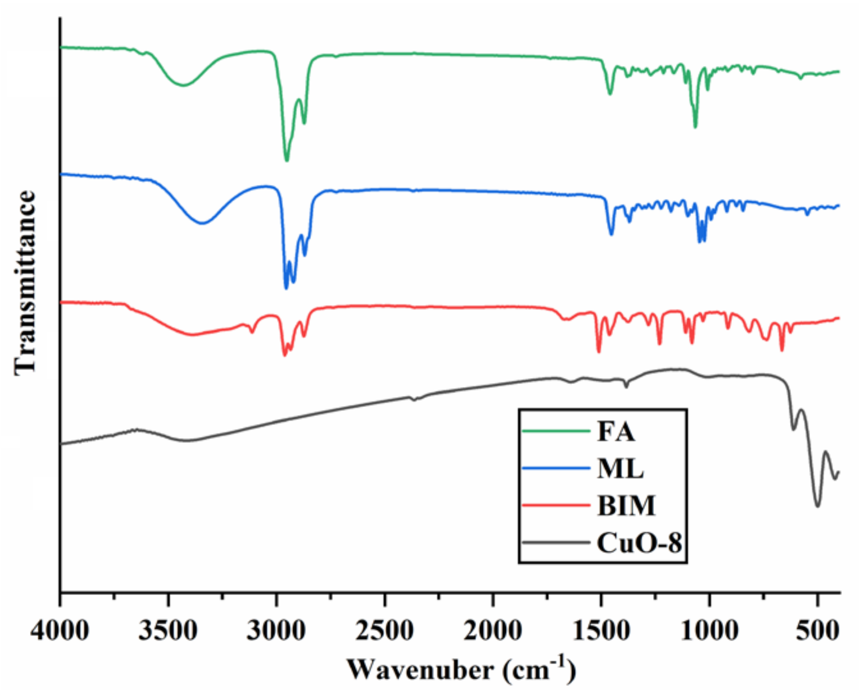

Figure S7. The FT-IR spectra of FA, ML, BIM and CuO-8 NPs.

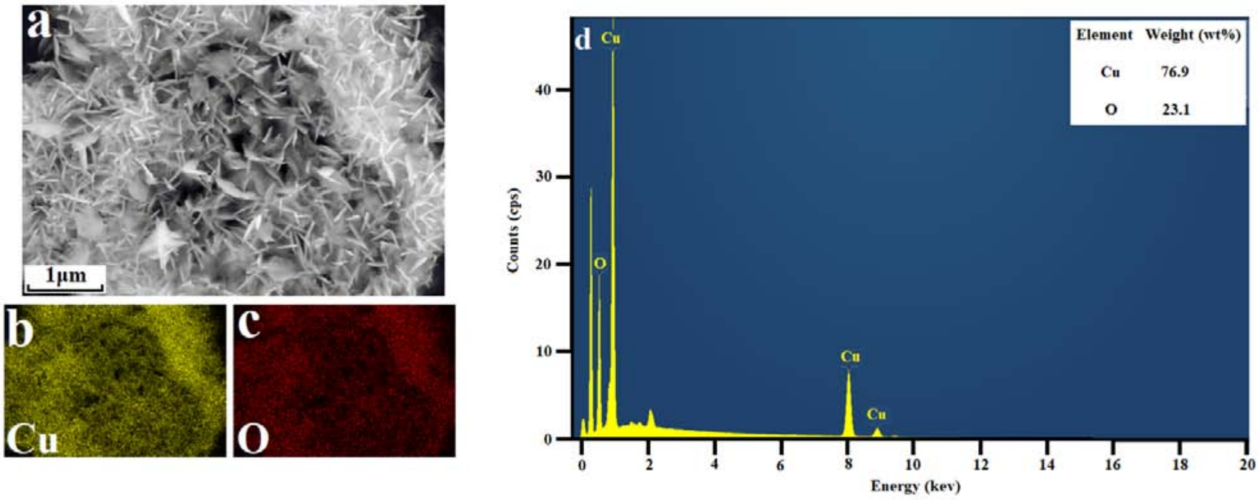

Figure S8. The SEM image (a), EDS mapping (b, c) and EDX pattern (d) of CuO-2 NPs.

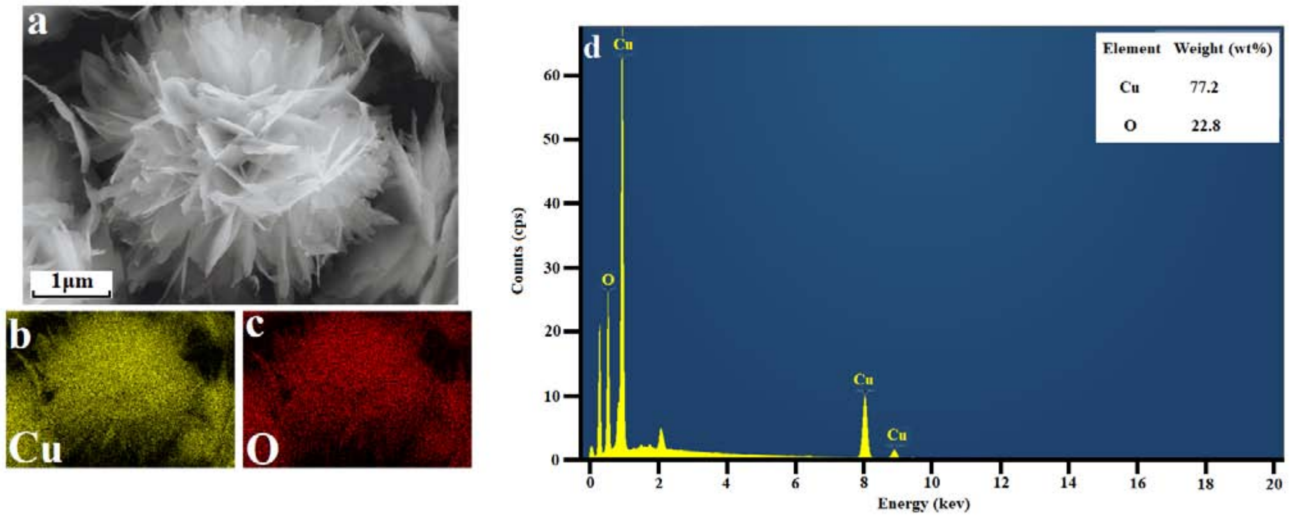

Figure S9. The SEM image (a), EDS mapping (b, c) and EDX pattern (d) of CuO-3 NPs.

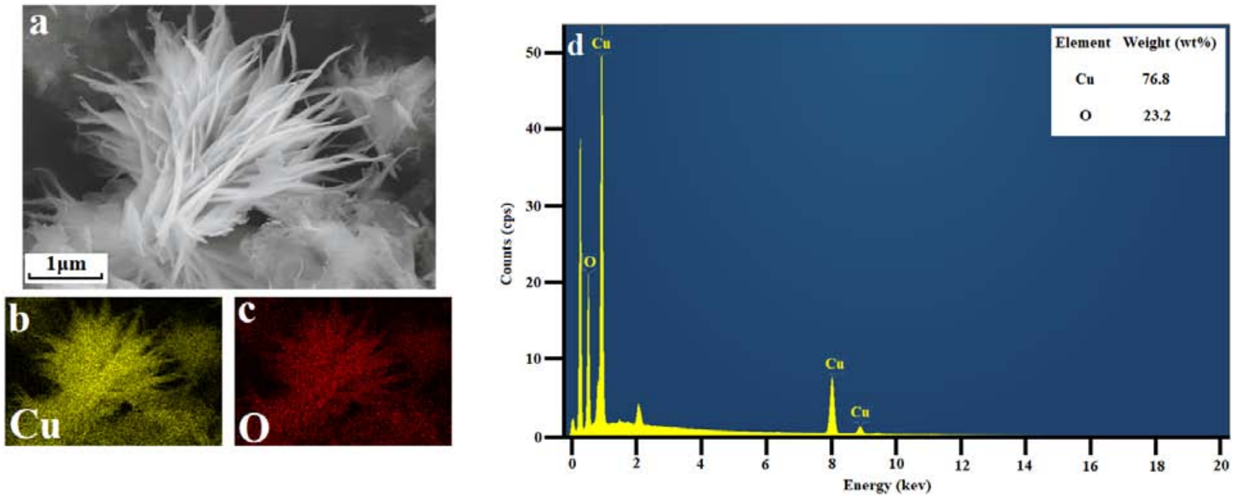

Figure S10. The SEM image (a), EDS mapping (b, c) and EDX pattern (d) of CuO-4 NPs.

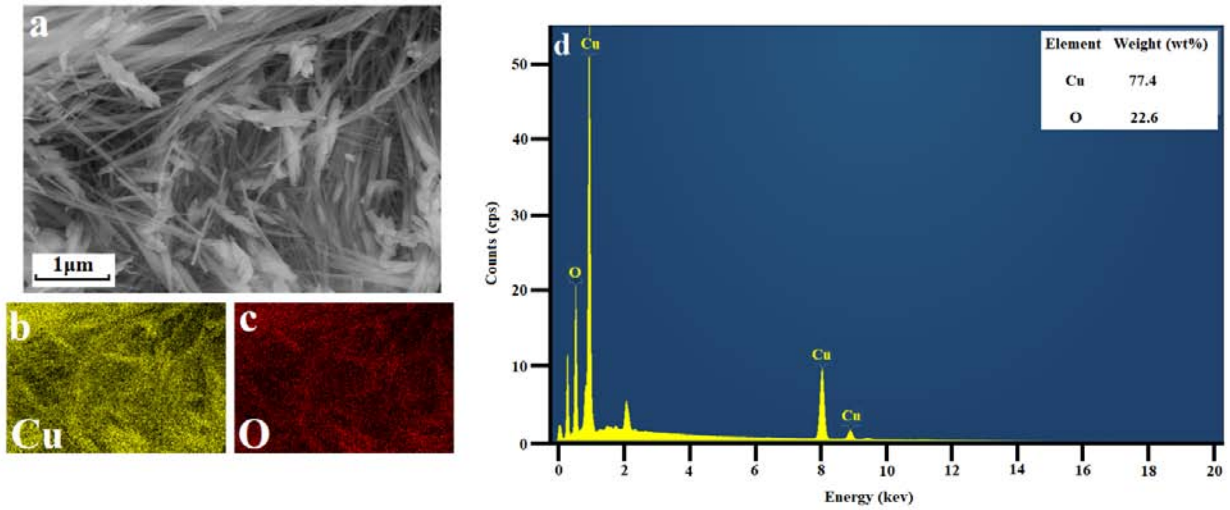

Figure S11. The SEM image (a), EDS mapping (b, c) and EDX pattern (d) of CuO-5 NPs.

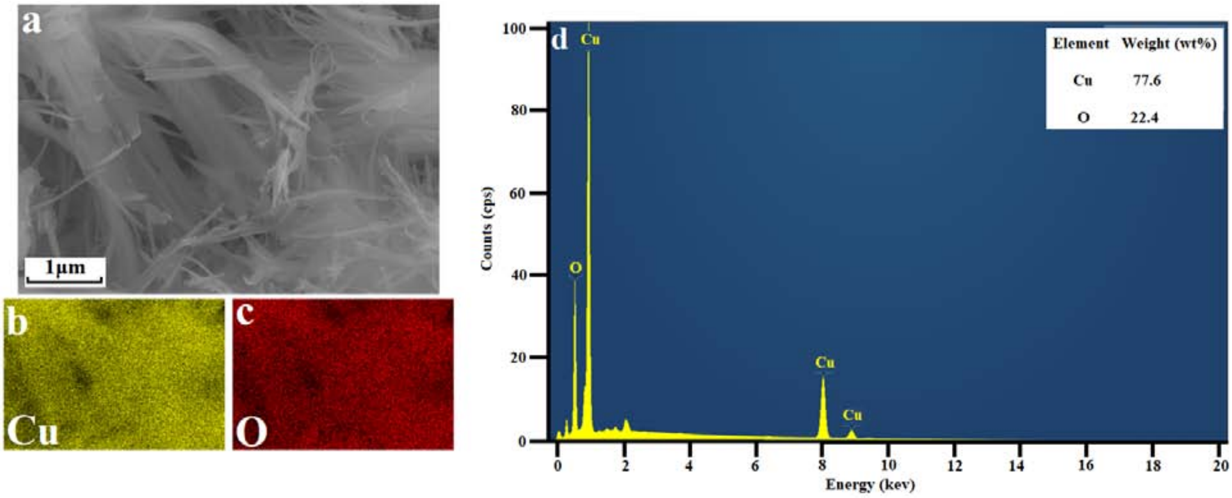

Figure S12. The SEM image (a), EDS mapping (b, c) and EDX pattern (d) of CuO-6 NPs.

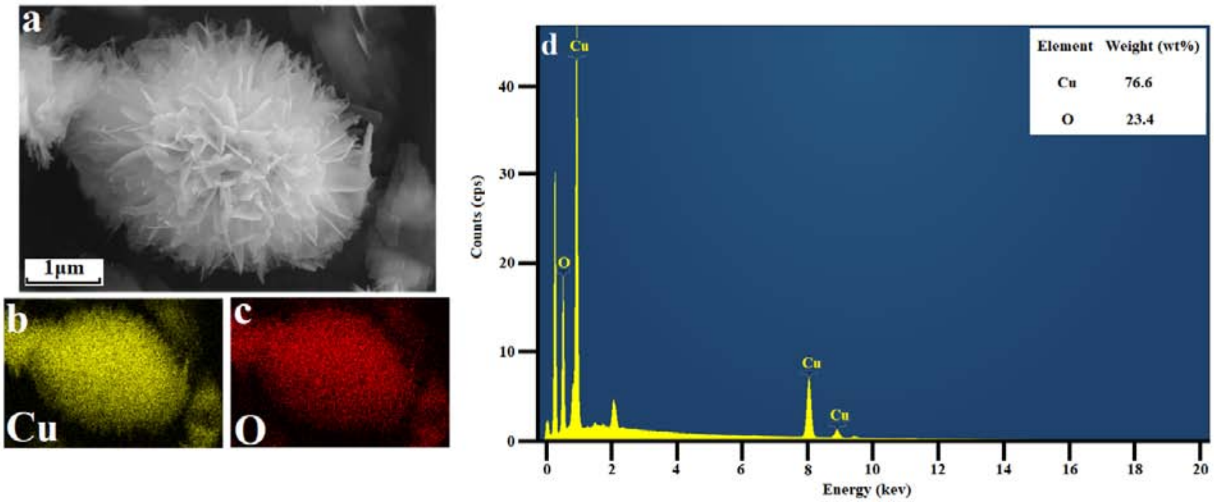

Figure S13. The SEM image (a), EDS mapping (b, c) and EDX pattern (d) of CuO-7 NPs.

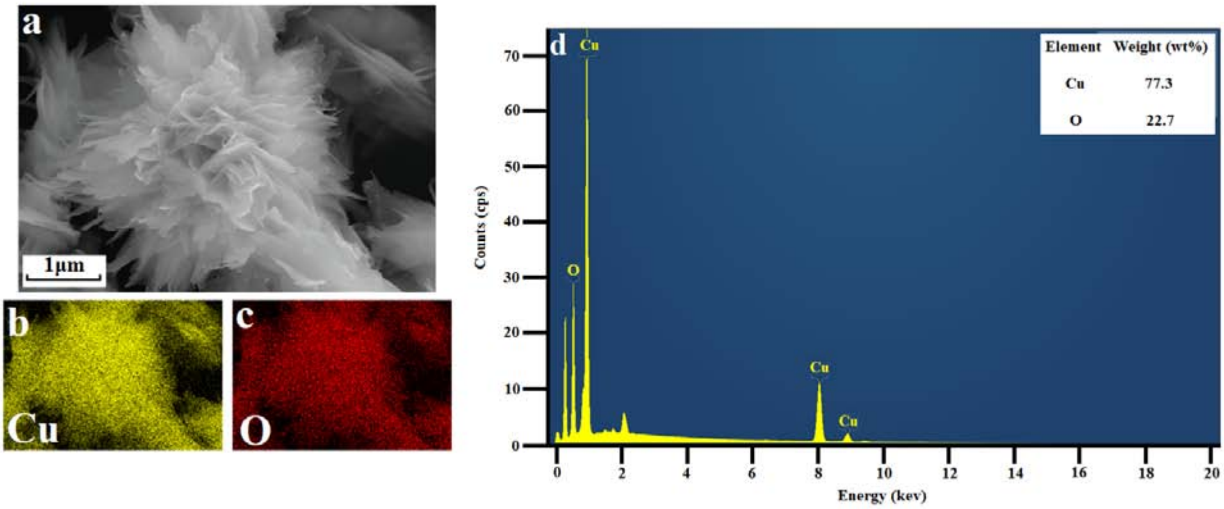

Figure S14. The SEM image (a), EDS mapping (b, c) and EDX pattern (d) of CuO-8 NPs.

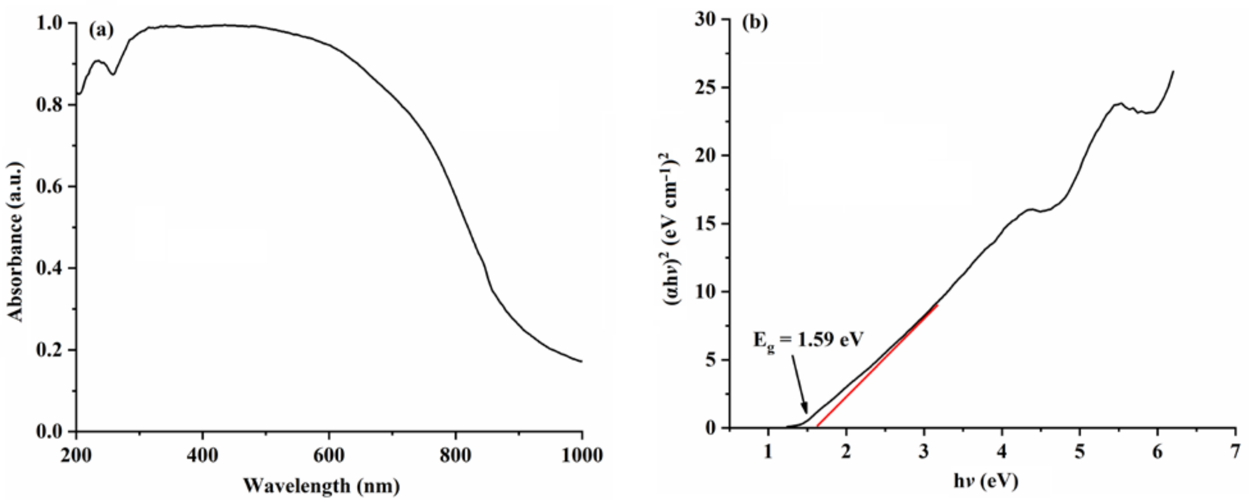

Figure S15. The UV–Vis absorption spectrum (a) and Tauc plot (b) of mesoporous CuO-2.

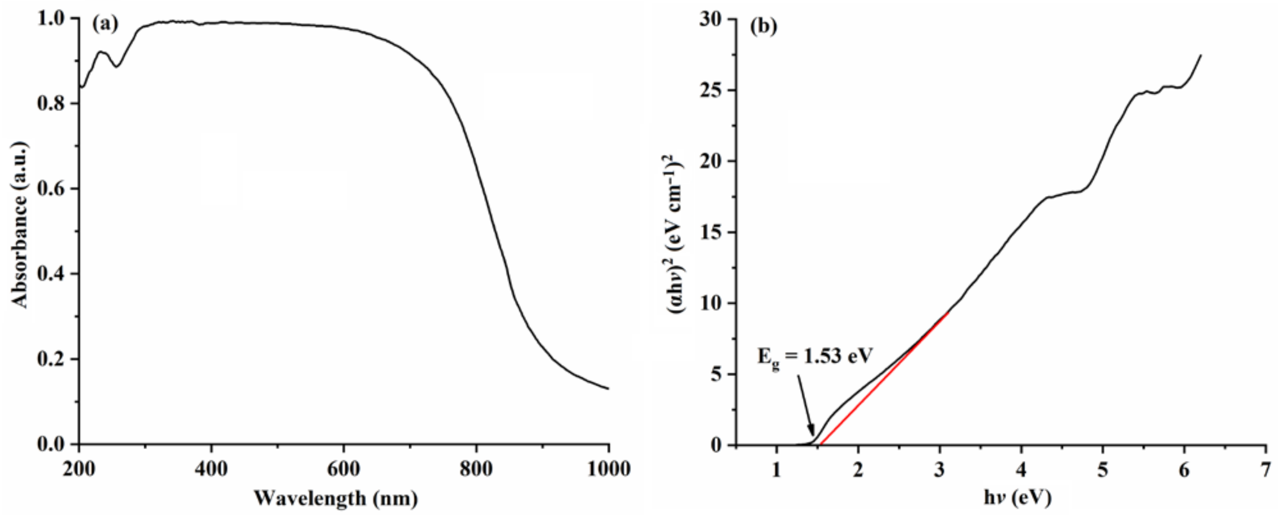

Figure S16. The UV–Vis absorption spectrum (a) and Tauc plot (b) of mesoporous CuO-3.

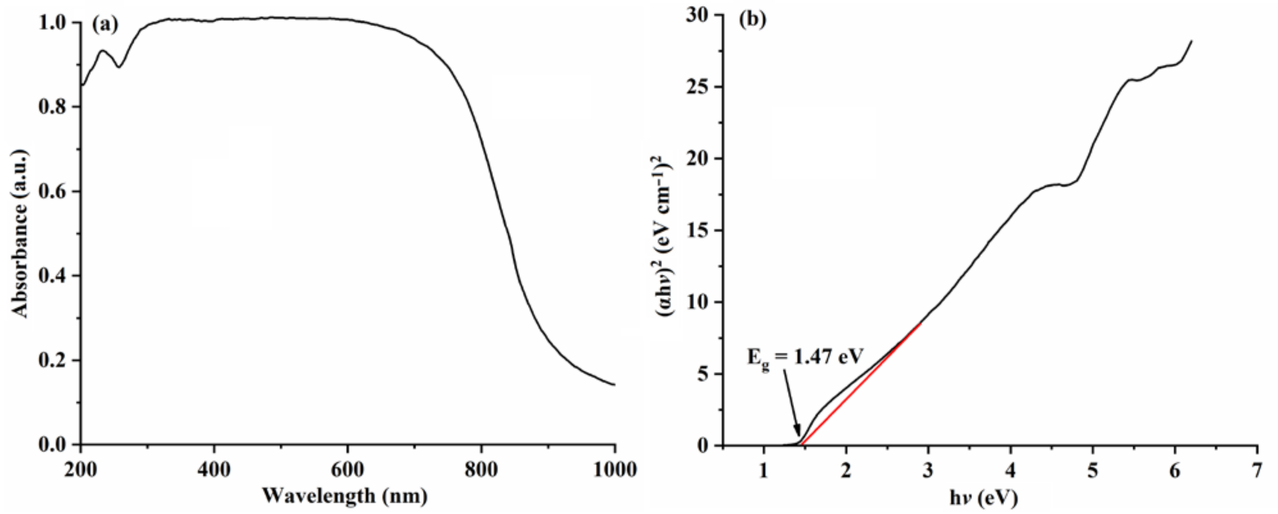

Figure S17. The UV–Vis absorption spectrum (a) and Tauc plot (b) of mesoporous CuO-4.

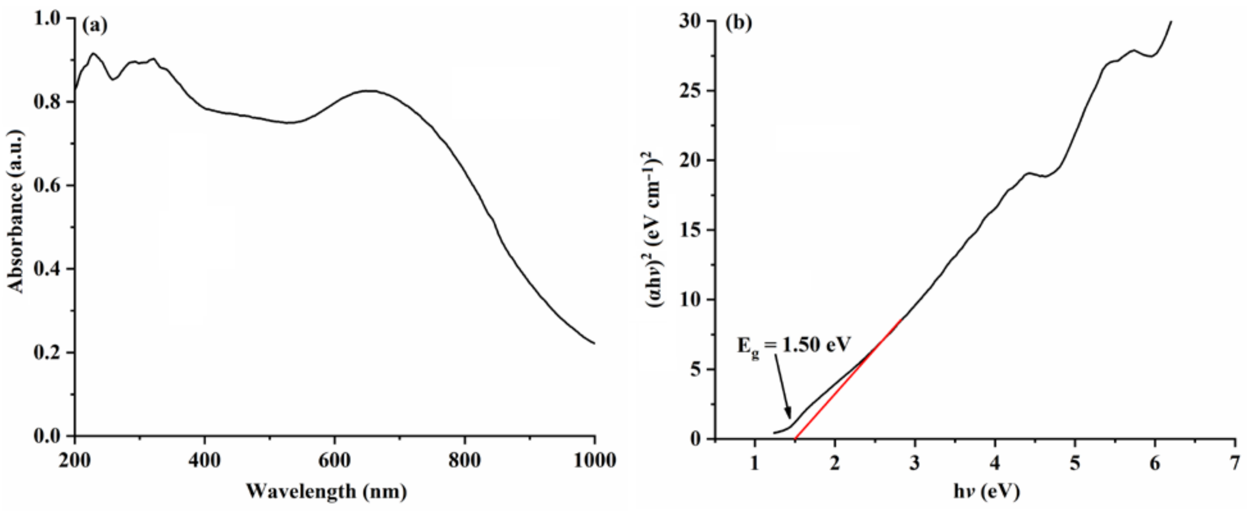

Figure S18. The UV–Vis absorption spectrum (a) and Tauc plot (b) of mesoporous CuO-5.

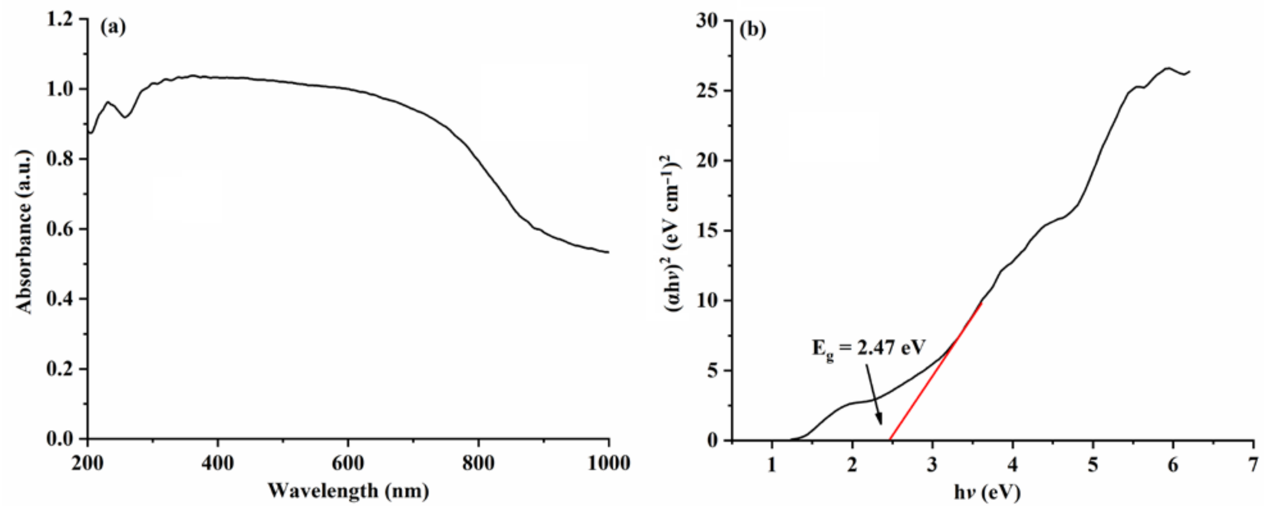

Figure S19. The UV–Vis absorption spectrum (a) and Tauc plot (b) of mesoporous CuO-6.

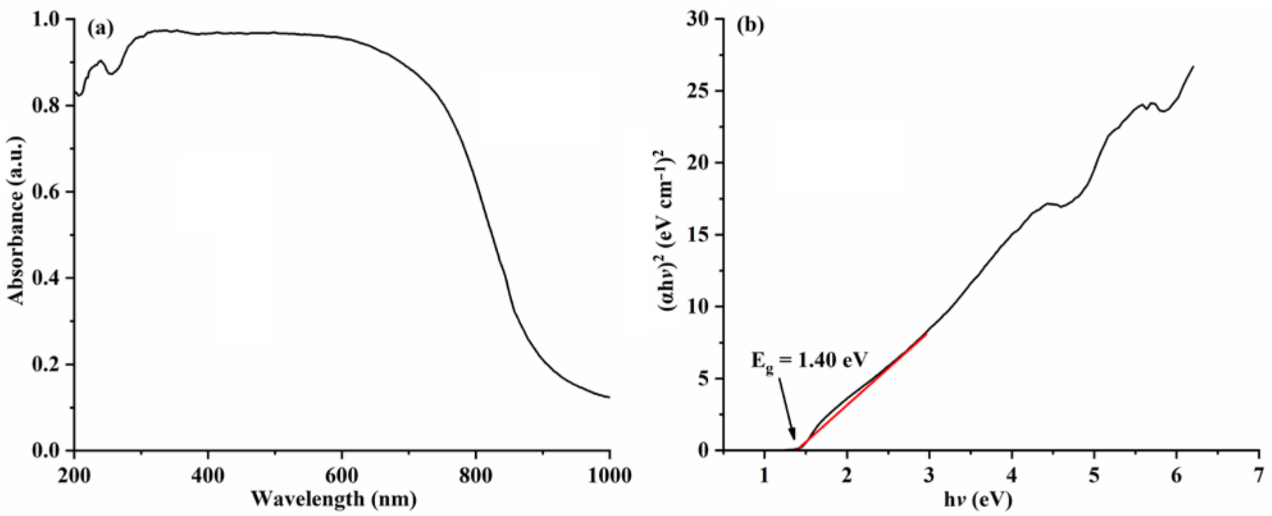

Figure S20. The UV–Vis absorption spectrum (a) and Tauc plot (b) of mesoporous CuO-7.

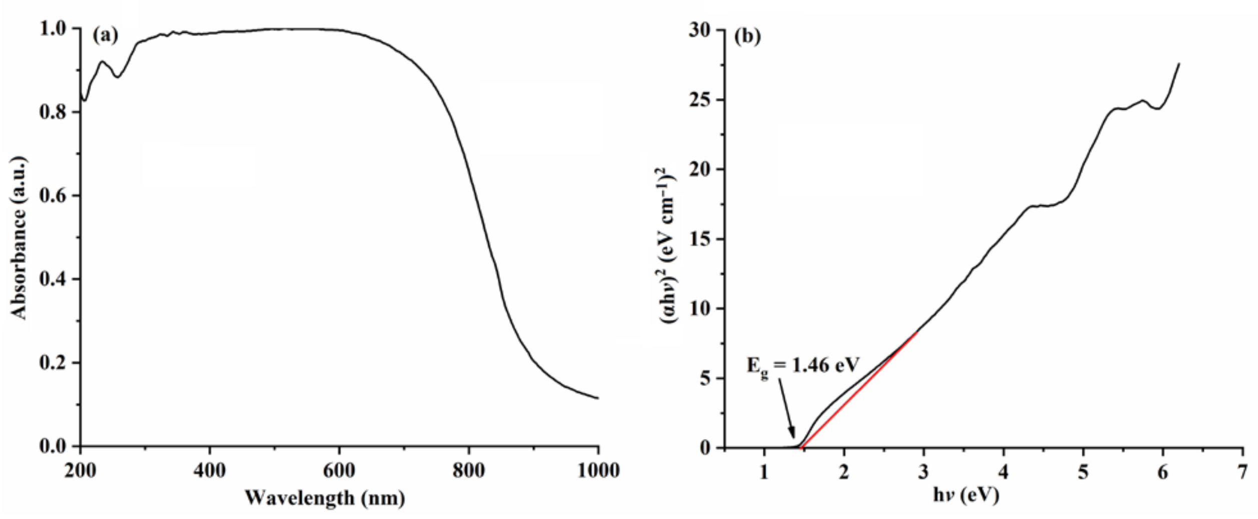

Figure S21. The UV–Vis absorption spectrum (a) and Tauc plot (b) of mesoporous CuO-8.

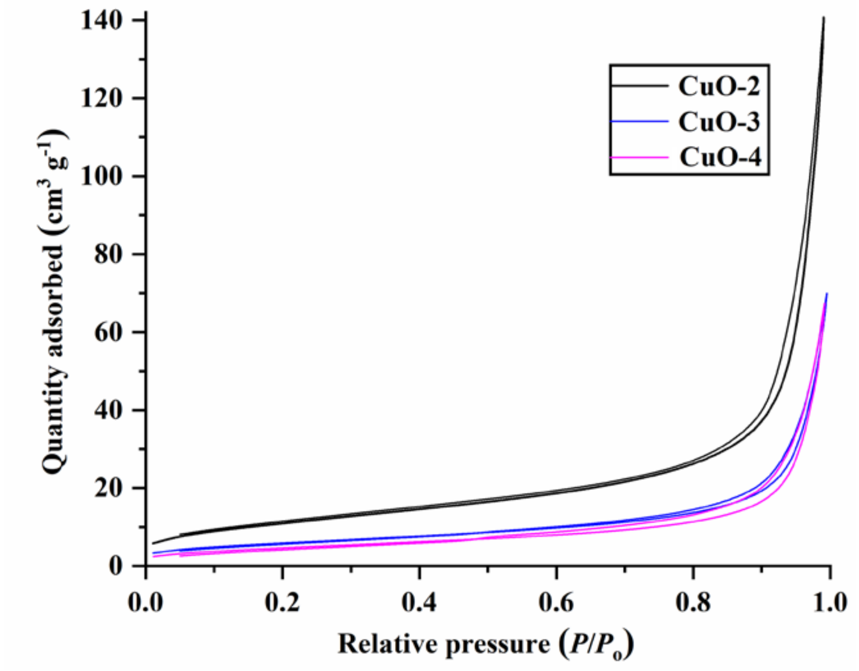

Figure S22. The N_2 adsorption–desorption isotherms (77 K) of the synthesized CuO-2, CuO-3 and CuO-4.

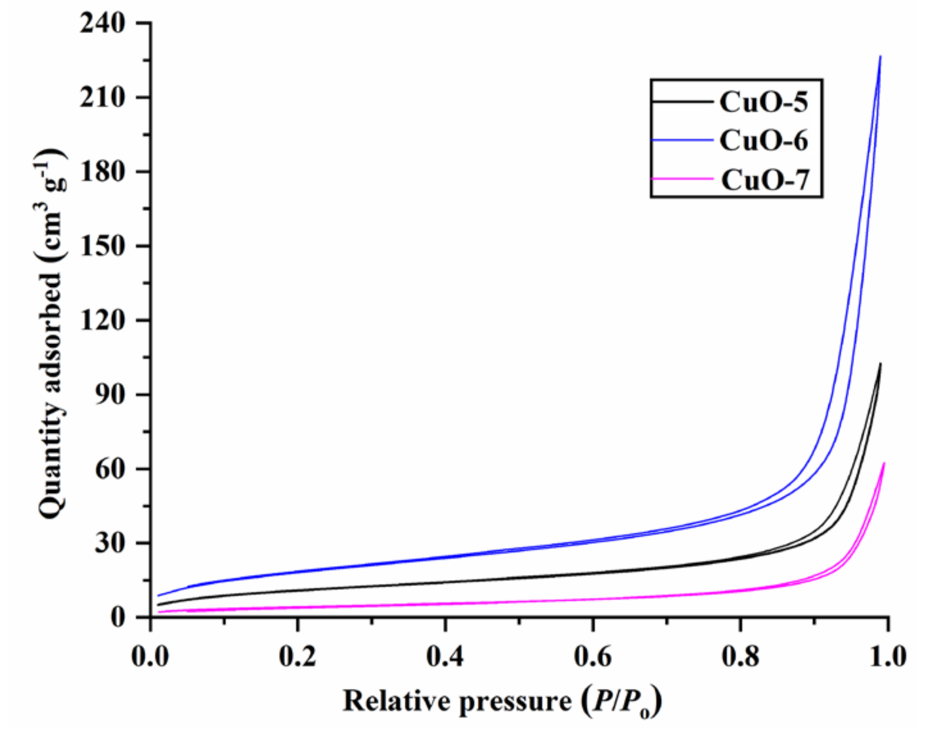

Figure S23. The N_2 adsorption–desorption isotherms (77 K) of the synthesized CuO-5, CuO-6 and CuO-7.

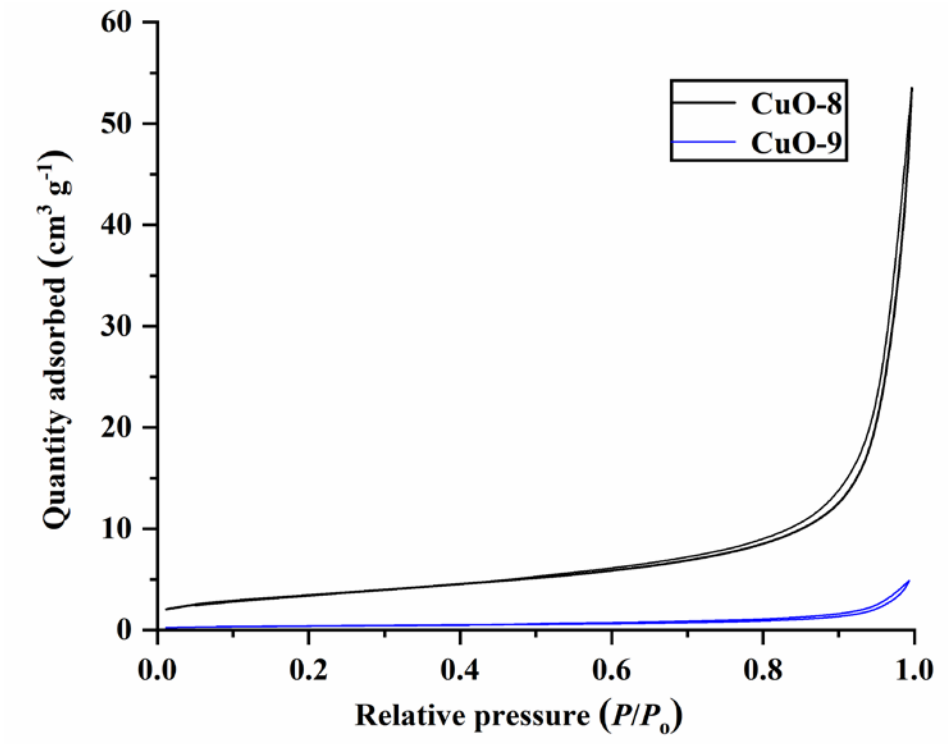

Figure S24. The N_2 adsorption–desorption isotherms (77 K) of the synthesized CuO-8 and CuO-9.
